# Epidemiology, Clinical Signs, and Risk Factors Associated with Theileriosis in Australian Cattle (2006–2022)

**DOI:** 10.3390/pathogens13030253

**Published:** 2024-03-15

**Authors:** Emily Onizawa, Cheryl Jenkins

**Affiliations:** 1 NSW Department of Primary Industries, Elizabeth Macarthur Agricultural Institute, Menangle, NSW 2568, Australia; emily.onizawa@dpi.nsw.gov.au; 2 Animal Science, School of Environmental and Rural Science, University of New England, Armidale, NSW 2351, Australia

**Keywords:** *Theileria orientalis* Ikeda, bovine anaemia, distribution, Australia, *Haemaphysalis longicornis*

## Abstract

For over a decade, bovine anaemia caused by *Theileria orientalis* Ikeda has been a significant disease in the Australian cattle industry. In this study, we conducted a spatial and temporal analysis of theileriosis in Australia using historic data from submissions to the New South Wales Department of Primary Industries (NSW DPI) from 2006 to 2022, where herd history, clinical signs, and PCR results were available. Since the first detections of bovine theileriosis in the Sydney area in 2006, the disease spread north- and southward and is now endemic to the southeast coast of Australia, closely mirroring the distribution of the principal vector *Haemaphysalis longicornis.* Across all years, the prevalence of the Ikeda genotype was 88%, while the prevalence of the benign Chitose and Buffeli genotypes was 55% and 38%, respectively. The majority of submissions were from beef cattle in coastal NSW, with anaemia, fever, jaundice, abortion, and lethargy the most frequently reported clinical signs. Transportation was identified as the major risk factor for disease. Until 2015, the majority of cases were reported in adult cattle, while in later years, calves made up the majority of cases, most likely due to the widespread acquisition of immunity in adults. Calves were significantly more likely to present with diarrhoea, lethargy, and anaemia, and to suffer mortality, while adults were significantly more likely to present with jaundice. Instances of abortion were observed to be significantly associated with beef cattle. The relationship between the level of parasitaemia and anaemia revealed a strong negative correlation for all animals examined.

## 1. Introduction

The haemoprotozoan parasite *Theileria orientalis* is a tick-borne piroplasmid that typically infects cattle. Distinguished from the highly pathogenic *Theileria parva* and *Theileria annulata* (the causes East Coast fever and tropical theileriosis, respectively), which occur in cattle in Africa, Europe, and Asia [1], *T. orientalis* was originally thought to be relatively benign [2]. *T. orientalis* has been detected on several continents, including Africa [3] and Europe [4,5]; however, disease outbreaks are mainly seen in parts of Asia [6,7], Oceania [8,9], and North America [10]. Outbreaks are typically associated with a pathogenic genotype of the parasite known as the Ikeda genotype (or Type 2) [11,12,13], although 10 other genotypes have been identified based on sequences of the major piroplasm surface protein (MPSP) gene [14]. Though most genotypes are considered non-pathogenic, outbreaks of clinical theileriosis associated with the Ikeda genotype have been on the rise in recent years with outbreaks in Australia [8], New Zealand (NZ) [15], and the United States (U.S.) [15,16]. Clinical disease associated with the Chitose genotype (Type 1) is observed less frequently [9] or in mixed infections with the Ikeda genotype [17,18]. 

In Australian cattle, Types 1–5 have been identified [14], with the most common genotypes being *T. orientalis* Ikeda, *T. orientalis* Chitose, and *T. orientalis* Buffeli (Type 3) [18]. The Buffeli genotype has been present in Australia for over 100 years [19], but given its low pathogenicity, only very occasional cases of clinical theileriosis have been reported [20,21]. *Theileria* infections only became associated with significant clinical disease in Australia in 2006, with eight separate reported cases of haemolytic anaemia diagnosed as theileriosis through blood smear examination and exclusion of other causes [8]. Thereafter, there were increasing reports of bovine anaemia caused by *T. orientalis.* MPSP gene sequence analysis revealed the presence of the Ikeda genotype, which had been associated with theileriosis outbreaks in Japan and Korea in the decades prior [13]. A surveillance study on Australian cattle herds in 2010–2011 revealed that the Ikeda genotype alone or in combination with the Chitose genotype was the causative agent of the outbreaks seen in Australian herds [22]. 

*Haemaphysalis longicornis* [20,23,24], a member of the Ixodidae family, is recognised as the major vector for *T. orientalis*. Commonly known as the Asian-longhorned tick or bush tick, *H. longicornis* is a significant worldwide pest found to be present on three continents and in at least 10 countries [25]. This tick species readily establishes populations in new areas of suitable habitat through parthenogenesis and is responsible for spreading a number of diseases of veterinary and human importance [26]. In NZ, *H. longicornis* is considered to be the vector for *T. orientalis* [9] and is the only tick present in the country that infests livestock [24], having been introduced in the late 19th or early 20th century from imported cattle [27]. In the United States (U.S.), *H. longicornis* was first identified outside of quarantine on a sheep in August 2017 [28]. Within a year, there was evidence of further spread, and it was found on multiple host species, including wildlife, domestic animals, and humans [29]. Concurrent with the spread of the vector, there were increasing reports of theileriosis in cattle across the same locations [30]. The importation and movement of cattle and other animal species between Japan, Australia, and the U.S. is believed to have led to the introduction of both the vector and the disease [10], with the subsequent establishment of clinical theileriosis throughout the country. Various other *Haemaphysalis* sp. have also been shown to be vectors of *T. orientalis,* such as *H. megaspinosa* and *H. douglasi* in Japan [2]. In Australia, historically, it was thought that the cattle tick, *Rhipicephalus australis*, was the vector for theileriosis; however, it is now known that *T. orientalis* is transmitted by *H. longicornis* [23], with *H. bancrofti* (wallaby tick) also a likely vector [31]. *T. orientalis* can also be transmitted mechanically via biting lice [32] or iatrogenic means [33], and a low rate of transplacental transmission has also been reported [34,35]; however, it is notable that the majority of clinical disease outbreaks are reported to occur within the known range of the vector tick or where tick density is highest [30,36]. 

Being a parasite that proliferates within erythrocytes, it is not surprising that anaemia is one of the most frequently cited clinical signs of theileriosis, along with related observations, such as mucous membrane pallor and jaundice. Clinical signs that have been reported around the world also include pyrexia, depression, inappetence, weakness, and reduced lactation [10,11,12,37,38]. In Australia, commonly cited clinical signs include, but are not limited to, anaemia, jaundice, lethargy, and abortions [8,13,22,31,39]. Subclinical infections are also common where parasitaemia can be present without clinical disease, including infection with *T. orientalis* Ikeda [40,41]. Therefore, determination of the parasite load and discrimination of the genotypes present are important for making a diagnosis of theileriosis. 

Clinical cases of theileriosis are still being diagnosed after more than 15 years of this disease in Australia. The New South Wales Department of Primary Industries (NSW DPI) has been involved in diagnostic testing for bovine theileriosis in Australia since the first clinical cases caused by *T. orientalis* Ikeda were reported. This study aims to collate information from clinical cases occurring between 2006 and 2022 and provide an overview of the epidemiology of this disease in Australia. 

## 2. Materials and Methods

### 2.1. Data Collection

Details of diagnostic submissions made to NSW DPI between 2008 and 2022 were extracted from the NSW DPI Laboratory Management Information System (LIMS), Sample Manager (Thermo Fisher Scientific, Waltham, MA, USA). Details of diagnostic investigations made prior to 2008 were derived from archived NSW DPI research records. Diagnostic cases were submitted from 6 Australian states (Table 1), with 91% of jobs coming from NSW. Cases from surveillance studies conducted between 2009 and 2011 were also examined and include distribution and significance studies of *T. orientalis* genotypes in the states of NSW, Queensland (QLD), and Victoria (VIC) [41,42]. Submitters were also encouraged to submit samples to the laboratory during this period if animals showed signs of anaemia.

Data collated for this study included property location, production type (beef, dairy cattle, or unknown), age (calf, adult, or unknown), clinical history, and test results. For the purposes of this publication, adult cattle are defined as being >12 months of age. For each submission, a binary scoring system was used to document the presence of common clinical signs.

### 2.2. Clinical Analysis

Samples received and tested between 2006 and 2022 included anticoagulated blood, clotted blood, and splenic tissue. DNA extraction was performed using either detergent-proteinase K treatment (DPK) [43], DNeasy Blood and Tissue Kit (Qiagen, Hilden, Germany), or MagMax Core Nucleic Acid Purification Kit (A32702; Thermo Fisher). All extracts were stored at −20 °C. Prior to 2015, a conventional PCR for *T. orientalis* detection was performed as outlined in Eamens (2012). Since 2015, qPCR has been performed on extracted DNA following in-house methods detecting genes for MPSP genotypes [18,44] and quantified using plasmid standards. The primers used for qPCR were MPSP-F and MPSP-R, and the probes were Pr-U, a universal probe for all genotypes of *T. orientalis*, Pr-I for the detection of Ikeda genotype, and Pr-Ca/Pr-Cb for the detection of Chitose A and B genotypes. These assays were run as a triplex assay, as described in [44]. Detection of the Buffeli genotype was via a singleplex assay with MPSP-B-F, MPSP-B-R primers, and Pr-B probe, as described in [18]. *Theileria* gene copies were then calculated based on the quantity of *Theileria orientalis* MPSP gene amplified and classified into low (<15,000 gene copies), moderate (>15,000 but <300,000 gene copies), or high (>300,000 gene copies) levels [44]. Gene copies per microlitre of packed erythrocyte (GC/uL PE) were calculated using the same methods as Bogema et al. [44], with a correction factor of −16% applied to MPSP gene copy numbers from DNA extracted through the DPK method [43]. Conventional PCR-positive extracts generated prior to 2015, where available, were retrospectively tested by qPCR in this study to obtain quantitative data on parasite load, as described above. 

Packed Cell Volume (PCV) results were collated from a combination of testing at EMAI, outsourced testing, or testing conducted by the submitting veterinarian. Individuals with a PCV < 24 were considered anaemic and ≥24 were considered normal. Whether animals had severe anaemia (PCV < 15), moderate anaemia (PCV 15–24), or a normal haematocrit (PCV > 24) was also considered. 

To determine which clinical signs were most commonly associated with theileriosis, a subset of cases (*n* = 1194) where both a clinical history was provided and a qualified veterinarian had made a diagnosis of theileriosis was examined. To investigate the relationship between anaemia and the level of parasitaemia, diagnostic cases, as well as PCR-positive cases from surveillance activities, which included data for both PCV and parasite load (as determined by qPCR), were examined (*n* = 903). A contingency table was also created using this subset. 

### 2.3. Mapping and Statistical Analysis

To investigate the epidemiology of clinical theileriosis in Australia, the submissions data were filtered to include only herds where the presence of *T. orientalis* Ikeda was detected. Mapping and animation were conducted using ArcGIS Pro 3.0.2. Data analysis and graphing were completed using Microsoft Excel Version 2303 and GraphPad Prism 4. RStudio 2022.07.2+567 was used for statistical analysis, including correspondence analysis (CA) and calculation of the χ2 statistic to observe the relationship between the rows and columns of the contingency table. The factor map was also generated using R, as well as odds ratio, two-sided *t*-tests, and proportion testing calculations. 

## 3. Results

### 3.1. Theileriosis Sample Submission Summary and Overall Trends

**Figure 1:**
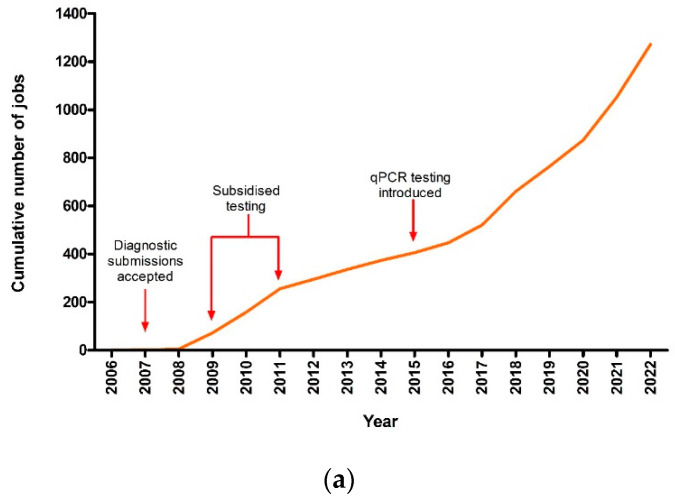

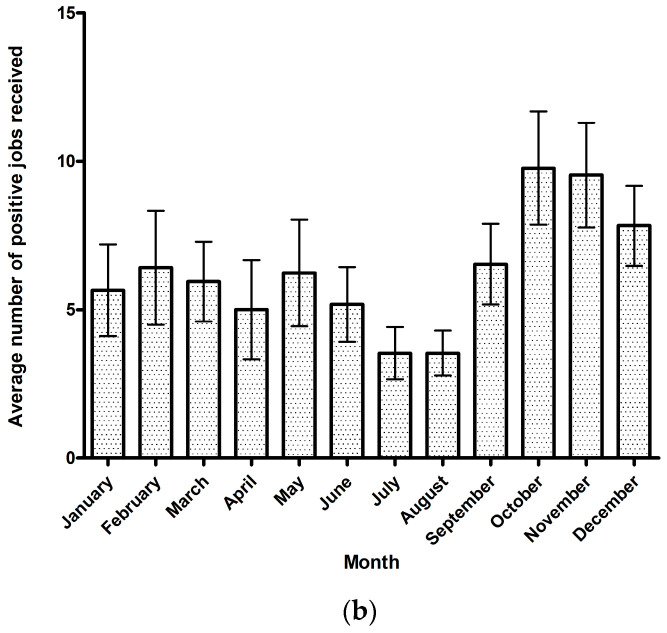
(**a**) Cumulative number of *Theileria*-positive jobs received at EMAI (**b**) and average number of submissions received each month for theileriosis testing from 2006 to 2022. Bars indicate standard error of the mean.

From 2006 to the end of 2022, there was a total of 1605 submissions received for *T. orientalis* testing. Each submission represented a single herd or property where various numbers of samples and sample types were submitted. Of these submissions, 79% returned at least one sample positive for *T. orientalis* by PCR. There was a spike in submissions between 2009 and 2011, where subsidised testing was offered as part of a theileriosis surveillance program; however, progressively, as the disease spread, each year, the number of submissions received and those that were positive for *T. orientalis* continued to increase (Figure 1a). By the end of 2022, there was a total of 265 jobs that had been submitted that year for *T. orientalis* testing, and of these, 220 jobs returned at least one positive PCR result.

While the number of submissions per month varied substantially from year to year, there was a seasonal trend in the average number of jobs received, with an increase in submissions for theileriosis testing between September and December (early spring to early summer) and the lowest average number of submissions from April to August (late autumn to end of winter) (Figure 1b). 

### 3.2. Breed and Age

The majority of the herds tested (70%) were beef herds, comprising *Bos taurus* cattle, including breeds such as Angus, Hereford, Limousin, or Charolais and their crossbred counterparts. This reflects the fact that the majority of cattle in Australia (over 90%) are bred for beef production and that a large proportion of beef production occurs in NSW (21%) [45], the state from which the majority of submissions were received. Samples from dairy cattle (almost entirely Holstein/Friesians) and either mixed or unknown farm types comprised the remaining 30% of submissions at 16% and 14%, respectively. The state of Victoria dominates the other states in dairy production, producing a substantially larger volume of milk compared to the other states [46], and is followed by NSW. 

**Figure 2:**
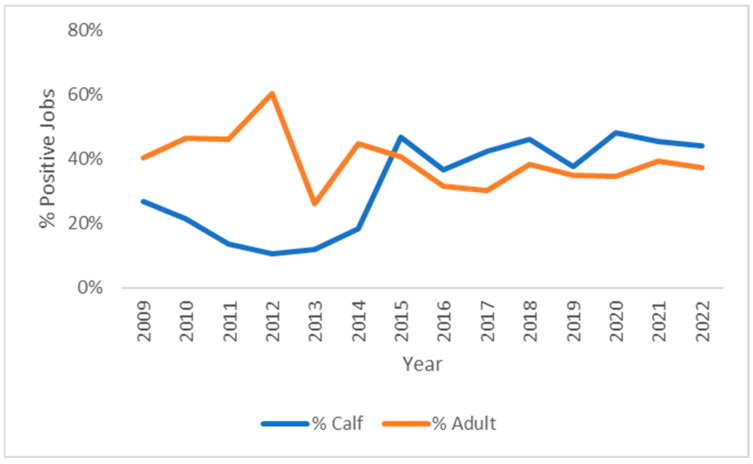
Percentage of jobs from adult cattle and calves between 2009 and 31 December 2022 that tested positive for *T. orientalis*.

Across all years, 39% of positive cases were in adults, 32% in calves, and 28% in cattle of unspecified age. However, since 2015, there has been a rise in calf cases, and the proportion of *Theileria*-positive cases in calves exceeded those in adult cattle (Figure 2).

**Figure 3:**
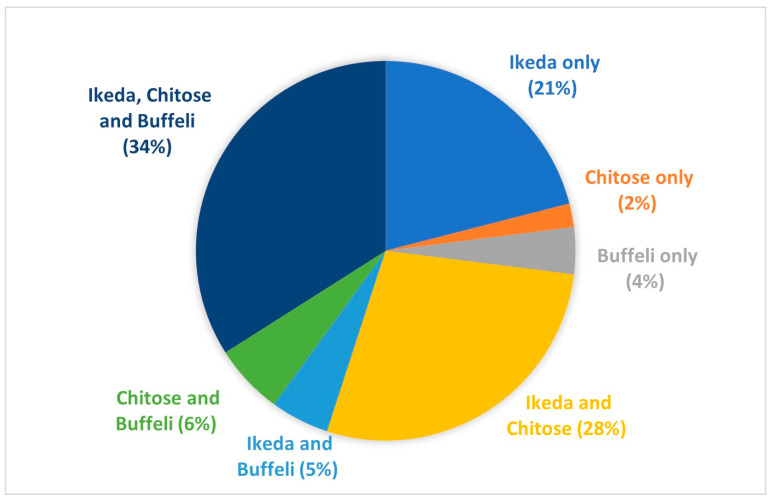
Total proportion of detected and genotyped jobs received at EMAI for theileriosis testing from 2008 to 31 December 2022.

Figure 3 shows the proportion of each genotype or mix of genotypes detected in herds from 2008 to 31 December 2022. Ikeda-only infections (21%), Ikeda and Chitose mixed infections (28%), or Ikeda, Chitose, and Buffeli mixed infections (34%) were the most common combinations observed. Herds with Chitose only (2%), Buffeli only (4%), or Chitose and Buffeli infections (6%) were relatively low in number; however, this is unsurprising, given that the animals examined were largely from diagnostic submissions. 

Across all submissions, *T. orientalis* was detected in 79% of cases, of which 88% were positive for the Ikeda genotype. This finding is similar to studies conducted in New Zealand, where up to 94% of herds in some regions were Ikeda-positive [47]. In contrast, the total prevalence of each of the benign genotypes in this study was 55% and 38% for Chitose and Buffeli, respectively.

### 3.3. Distribution of Clinical Theileriosis in Australia

**Figure 4:**
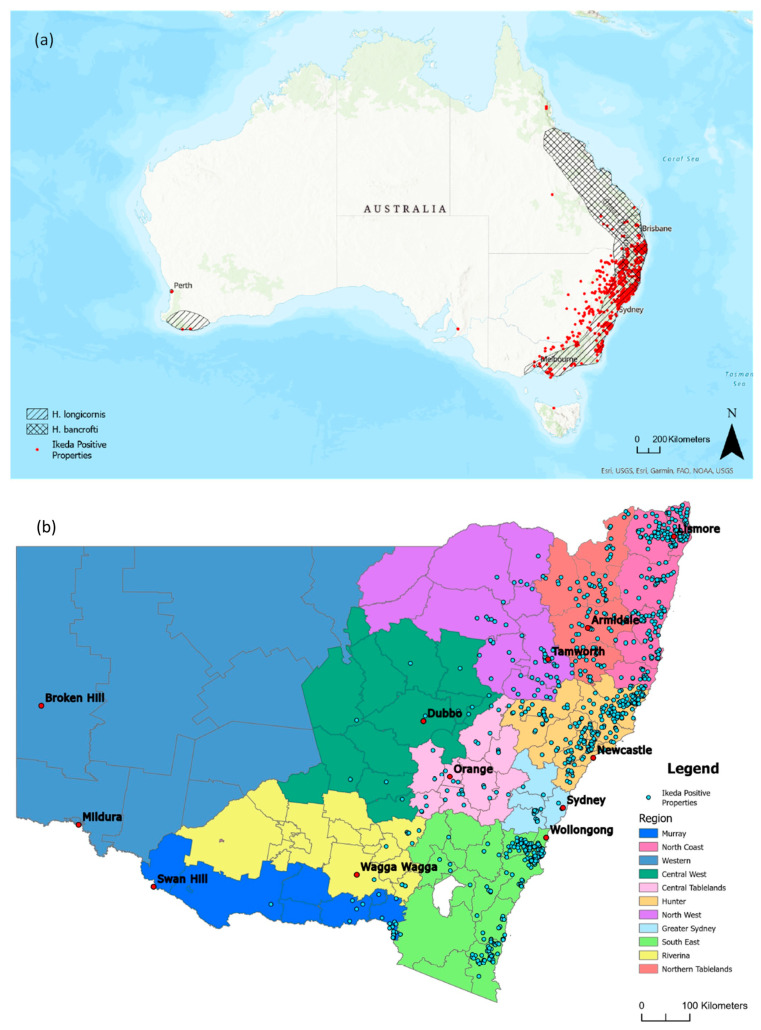
(**a**) All properties with theileriosis (*T. orientalis* Ikeda) cases detected between 2006 and 2022 (red dots) overlaid with the known ranges of *Haemaphysalis longicornis* and *Haemaphysalis bancrofti* [23,49], (black diagonal and black checked shading, respectively) and (**b**) map of NSW showing all Ikeda-positive properties (blue dots) and the Local Land District boundaries. NSW Local Land Service Region dataset: © State of New South Wales (Spatial Services, a business unit of the Department of Customer Service NSW).

The animation in Video S1 displays the number and location of new Ikeda-positive herds detected every month between 2006 and 2022. Following the initial cases detected around the Sydney area in 2006, *T. orientalis* Ikeda spread along the north coast of NSW and subsequently along the south coast, reaching the states of QLD in 2009 and VIC in 2010. Also in 2010, cases were detected in the far north of QLD in the Atherton Tablelands region. Positive detections continued along the eastern seaboard from southeast QLD to southern VIC, appearing in the southwest of WA in 2013 [39]) and southeastern SA in 2014 [48]. After 2015, the geographical spread of *T. orientalis* Ikeda largely ceased, with new positive detections occurring within areas where the parasite was already endemic. The data used in this animation were also overlayed with known distributions of *H. longicornis* and *H. bancrofti* (Figure 4a) based on a previous publication [49]. Properties with cattle testing positive for the Chitose and Buffeli genotypes were also found to occur across the same range as Ikeda-positive properties, although the full range of occurrence of these genotypes is unclear, given that Buffeli and Chitose are considered benign genotypes and the majority of submissions examined in this study were from clinical cases. 

The distribution of Ikeda-positive properties was also examined in more detail in NSW across Local Land Districts, given that the majority of clinical submissions were from this state (Figure 4b). Although Ikeda-positive properties were detected in the North and Central West as well as the Riverina and Murray districts, which are located west of the Great Dividing Range, the majority of positive detections occurred in coastal areas and areas on the eastern slopes of the Great Dividing Range, particularly the North Coast, Hunter, and South East LLS regions. The spread of theileriosis within NSW over time is shown in detail in Video S2.

### 3.4. Clinical Signs and Parasitaemia

**Figure 5:**
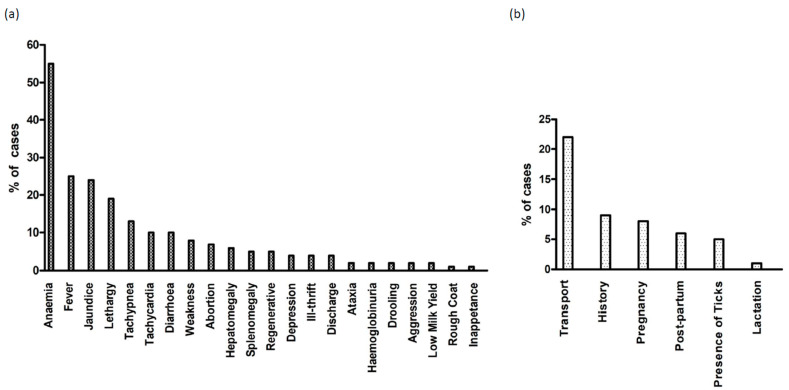
Percentage of theileriosis cases with (**a**) the 22 most commonly reported clinical signs and (**b**) the 6 major risk factors for disease total cases examined (*n* = 1195).

To determine the major clinical signs associated with theileriosis, a total of 1195 cases where the clinical history had been documented were examined. The top five signs reported in association with theileriosis included anaemia (*n* = 658), fever (*n* = 298), jaundice (*n* = 287), lethargy (*n* = 227), and tachypnoea (*n* = 157) (Figure 5a). In this instance, anaemia was recorded as a reported sign if the term was used by the submitting veterinarian, independent of any measurement of PCV. Terms implying anaemia, such as “pale mucous membranes” or “watery blood”, were also characterised as anaemia. Under this definition, anaemia was consistently the highest reported clinical sign for both calves (65%) and adults (51%). Fever (30%), lethargy (25%), jaundice (21%), and tachypnoea (18%) were the next most common signs reported for calves, while jaundice (28%), fever (24%), lethargy (17%), and abortion (13%) were the next most common reported in cows. Anaemia (55%), fever (28%), jaundice (25%), lethargy (19%), and tachypnoea (14%) were the top reported signs for beef cattle and anaemia (63%), jaundice (22%), lethargy (19%), fever (19%), and tachypnoea (13%) for dairy cattle.

The most commonly reported risk factors for theileriosis are summarised in Figure 5b. Transportation was the highest reported risk factor, with 22% (261/1195) of cases involving the transportation of animals within the preceding 6 months, either through purchases or agistment. Where transportation was a reported risk factor, 50% of movements involved the transit of cattle between endemic and non-endemic areas. Properties with a history of theileriosis were at risk of recurrent outbreaks, with 9% (105/1195) of cases citing a prior history of the disease. Interestingly, while ticks are the primary vector for theileriosis, only 56/1195 cases (5%) reported observations of ticks on affected cattle.

**Figure 6:**
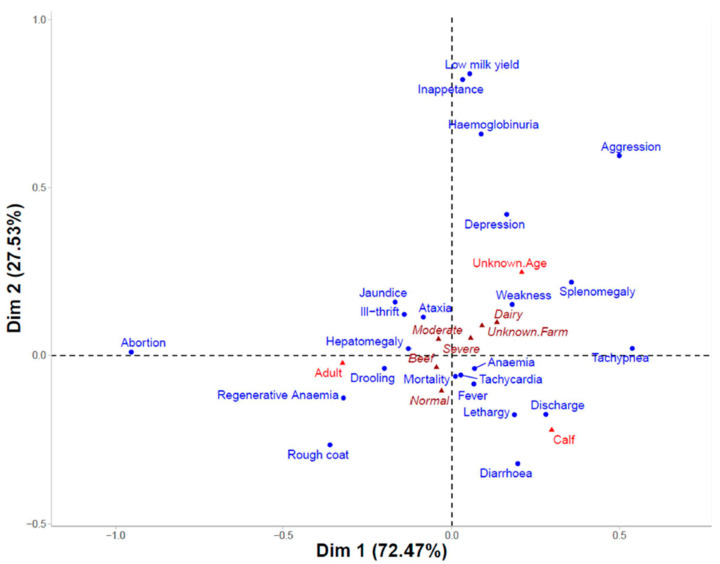
Factor map from correspondence analysis (CA) performed on *Theileria orientalis* Ikeda-positive individuals with clinical signs (blue) with variables of age category (red triangle) and supplementary categories of farm type and level of anaemia (dark red triangles).

A contingency table based on 903 individuals and listing the 23 major clinical signs (rows) and 12 columns, including the totals for the categorical variables age, farm type, and level of anaemia (Table S1), was created to perform correspondence analysis (CA) to investigate any associations between the clinical signs and the categories of age, level of anaemia, or production type. The resulting factor map is shown in Figure 6. An χ^2^ test (χ^2^ = 256, *p* < 0.0001) demonstrated that there was a strong association between the rows and columns of the contingency table. This was also supported by the correlation coefficient (0.34) calculated by the square root of ϕ^2^ (trace), where significant dependency is indicated when trace > 0.2 [50]. 

Odds ratios (ORs) were calculated to determine whether individual clinical signs were more likely to occur in calves vs. adults or in beef vs. dairy cattle. Clinical signs that were statistically different between groups are reported in Table 2. Calves had greater odds of presenting with diarrhoea, discharge, fever, and anaemia, as well as other signs related to anaemia, including lethargy and tachypnea. Calves were also more likely to suffer mortalities. In contrast, adult cattle were more likely to present with jaundice. Significant differences in clinical signs were also observed depending on production type, with beef cattle much more likely to experience abortion as well as tachycardia, fever, and mortality. Dairy cattle were more likely to suffer ill-thrift.

**Figure 7:**
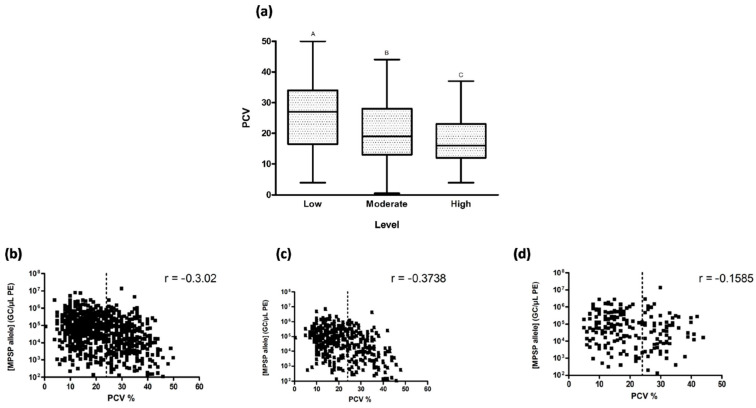
(**a**) PCV values from animals with (<15,000 gene copies), moderate (>15,000 but <300,000 gene copies), or high (>300,000 gene copies) levels of *T. orientalis,* as defined in [44], where the thick middle line represents the median value and lower and upper bars represent the minimum and maximum values, respectively. Subscripts A, B and C indicate statistical significance between levels of parasitaemia (*p* < 0.0001) (**b**–**d**) Correlation of gene copies per microlitre of packed erythrocyte (GC/µL PE) with PCV for (**b**) all individuals, (**c**) adults only, and (**d**) calves only. The vertical dotted line represents PCV of 24 where anaemia is classified when PCV < 24.

When observing the relationship between PCV and the level of parasitaemia, clinical anaemia (i.e., PCV < 24) was more common in animals with higher parasite loads (Figure 7a), and there was a significant difference (*p* < 0.0001) between each level of low, moderate, and high parasitaemia, as defined by Bogema et al. [44].

Within the high parasitaemia category, 77% of animals presented with anaemia, compared to 63% and 40% in the moderate and low categories, respectively. In addition, 44% of the individuals with high parasitaemia had severe anaemia (PCV < 15). Spearman’s rho indicated a relatively strong negative correlation between PCV and GC/µL PE when all animals were considered (Figure 7b) and when adult cattle alone were examined (Figure 7c). However, when calves alone were examined, the negative correlation between PCV and GC/µL PE was only moderate (Figure 7d). Nonetheless, in all cases, the negative correlation was significant (*p* < 0.0001).

## 4. Discussion

This study presents comprehensive cumulative data on the geospatial distribution and temporal spread of *T. orientalis* Ikeda in Australia. The disease is presumed to have first been introduced into Australia with the Ikeda genotype, probably via live cattle imported to establish the Wagyu breeding line in the early 2000s [51], as screening of cattle for *T. orientalis* was not a pre-requisite for importation. Infections with *T. orientalis* are lifelong, and given the presence of suitable disease vectors (*Haemaphysalis* spp. ticks) in Australia, it is perhaps unsurprising that theileriosis quickly became established. Following initial case reports between 2006 and 2008 on the mid-coast of NSW [8], which were later confirmed to be linked to the Ikeda genotype [13], there was a rapid increase in detections of theileriosis in NSW [13,23,39,41,52,53,54,55]. The high prevalence of the Ikeda genotype (88% of *T. orientalis*-positive submissions) is comparable to studies conducted in New Zealand, where up to 94% of herds in some regions were Ikeda-positive [47]. Theileriosis spread rapidly north- and southward along the coastal fringe of NSW from 2008 onward, with positive cases confirmed in southeast QLD and northern VIC in 2010. SA had its first confirmed case in 2012 and WA in 2013, with sporadic cases since then. *T. orientalis* Ikeda has not yet been detected in Tasmania, the Northern Territory (NT), or the Australian Capital Territory (ACT). The rate of spread can be attributed to the transport of cattle and the associated mixing of naïve and infected animals, which was identified here as the major risk factor for disease. 

Between 2015 and 2022, there appears to have been no further spread of *T. orientalis* Ikeda into new geographical areas, although cases of theileriosis continue to occur within the endemic zone. Herds within the endemic zone have been shown to rapidly reach a high infection prevalence once *T. orientalis* Ikeda is introduced [53] and subsequently enter a carrier state where subclinical infection is maintained. This carrier state appears to protect adult cattle against the development of clinical disease unless stressors, such as parturition or transport stress, induce recrudescence. This widespread acquisition of immunity within adult cattle limits the number of clinical cases observed. By 2015, endemic stability (where infection prevalence is high, but clinical cases are low) had likely been reached across the majority of the endemic zone. Around this time, the proportion of cases seen in calves relative to adult cattle increased substantially. A likely reason for this is that neonatal calves have little or no protection against theileriosis through maternal immunity. The colostral transfer of antibodies from dams to calves is both inconsistent and insufficient to prevent calves born in endemic areas from becoming highly parasitaemic within a few weeks of birth [33,35]. Thus, in more recent years, the majority of clinical cases from *Theileria*-endemic areas are seen in young, susceptible calves rather than adult cattle.

The geographical distribution of theileriosis appears to be limited by the range of the main disease vector, *H. longicornis*. In Australia, the known ranges of *H. longicornis* include the cooler, wetter environments along the lower half of the east coast of Australia, spanning down to Victoria [56,57]. There have been no comprehensive studies of the range of this vector since 1970; however, given the invasive nature of *H. longicornis* [26,28], it would be unsurprising if the distribution of this tick had expanded in the last 50 years. Indeed, recent Australian studies that mention *H. longicornis* distribution in relation to the spread of theileriosis confirm that *H. longicornis* is present in the southwest corner of Western Australia [39,58] and the Limestone Coast of SA [48]. Cases of theileriosis observed in Far North QLD have been confined to the Atherton Tablelands. While this area is in a subtropical latitude rather than the more temperate regions favoured by *H. longicornis* and has not yet been documented as being within the range of this tick vector, the elevation (and therefore cooler climate) of the Tablelands likely makes this area suitable habitat for this species. 

While *H. bancrofti* has been posited as a vector for *T. orientalis* Ikeda [31,59], relatively few cases of theileriosis have been diagnosed within the range of this species that do not also overlap the range of *H. longicornis* [56,60]. In one study, observations of *H. bancrofti* but not *H. longicornis* in the Northern Tablelands of NSW, where theileriosis is known to occur [31], seem to suggest that *H. bancrofti* is also a vector for *T. orientalis* Ikeda. However, it is important to note that in that study, *H. bancrofti* ticks were only obtained from a single property. Furthermore, there is little evidence that this species is an efficient vector for transmission, given the lack of reported cases of theileriosis within the range of this vector, such as on the central coast of QLD. In our current study, ticks were only infrequently reported (5%) on properties affected by theileriosis. This could be explained by the fact that *H. longicornis* spends much of its lifecycle on pasture [58] and that questing activity appears to be nocturnal [61]. While some historical experimental transmission studies indicated that *H. bancrofti*, not *H. longicornis*, was a competent vector for *T. orientalis* [62,63,64], *H. longicornis* was recently shown to be a definitive host for *T. orientalis* Ikeda in Australia [61]. The discrepancy in results from these experimental transmission studies is best explained by variation in vector competence for the different genotypes of *T. orientalis*, given that the earlier studies were conducted with *T. orientalis* Buffeli. Indeed, differences in vector competence should be expected, given that *T. orientalis* genotypes are genetically distinct at the species level [65]. 

Nonetheless, given that positive cases have been detected outside of the known range of *H. longicornis,* it is possible that transmission may occasionally involve other vectors. While mechanical transmitters of *T. orientalis* have been identified, including other arthropods, such as lice, mosquitoes [23], tabanid flies, and midges [66], and iatrogenic modes of transmission through husbandry practices are known to occur [33], these transmission mechanisms do not support the *T. orientalis* lifecycle and do not result in clinical disease [67]. Therefore, alternate tick species or even low densities of *H. longicornis* are more likely responsible for these cases. The majority of cases of theileriosis occurring outside the known range of *H. longicornis* are on the Western slopes of the Great Dividing Range. While this area may be too hot and dry for *H. longicornis* populations to become fully established, local conditions during sufficiently cool and wet years may allow for ticks introduced on cattle from coastal areas to reproduce sufficiently to contribute to disease spread. 

While the number of submissions received each month varied substantially from year to year, there was a general trend showing a peak in submissions between September and December. One explanation for the increase during this period could relate to the lifecycle of *H. longicornis,* where nymphs and adults feed on the host to complete their lifecycle during spring and summer [23]. In Australia, joining occurs in October and November [46], when there may be an increased number of properties with purchases or agistment of introduced animals, which may also account for the seasonal peak in clinical submissions. 

In this study, the top five clinical signs associated with confirmed theileriosis cases were anaemia, fever, jaundice, lethargy, and tachypnoea. This is consistent with commonly reported signs from other countries such as Japan, Korea, NZ, and the U.S., where anaemia, fever, and weakness have been noted [10,11,12,37,38]. In an examination of 605 theileriosis cases in New Zealand, Lawrence et al. [36] noted jaundice as the most commonly reported clinical sign; however, that study considered pale mucous membranes and anaemia as separate clinical observations. That study also noted that jaundice was reported significantly more often in adults compared to calves, a finding that was supported by our data. The fifth most common clinical sign of “off milk” (19%) observed in the Lawrence et al. study was not commonly observed in our study, partially due to the fact that more dairy cases were examined in the New Zealand study (44% of submissions) compared to only 16% of dairy submissions received in this study. In Australia, theileriosis cases have often been reported with abortion listed as a clinical sign, including the first cases reported in NSW, Victoria, South Australia, and Western Australia [8,52]. Indeed, in this study, we found that 58/459 or 13% of cases involving adult cattle listed abortion as a clinical sign. Interestingly, Lawrence et al. (2017) found that abortions are reported much more rarely in association with theileriosis cases in New Zealand (4/605 submissions; 0.7%). An effect of breed or production type may be one explanation for these differences, given that the majority of cases examined in the New Zealand study were from dairy herds, while the majority of cases examined here were from beef herds. Indeed, beef cattle were shown here to have significantly greater odds (OR 6.40; *p* < 0.0001) of presenting with abortions compared to dairy cattle. Nonetheless, there may be factors other than breed or production type involved, given that abortion was still associated with 4% of cases in dairy cattle in this study, almost six times the proportion reported in New Zealand [38]. Seasonal alignment of vector activity with calving and lactation in New Zealand has been proposed as one reason for the lack of observed abortions and may also explain the increased observations of milk drop. Conversely, in Australia, year-round calving is more likely to leave cows exposed to *Theileria* infection during gestation [68]. Regardless, abortion as a feature of disease would be expected to decline in herds within endemic areas due to the acquisition of immunity in adult cows. 

There was a stronger association, with statistical significance (*p* < 0.001), between diarrhoea and calves compared to adults in the correspondence and OR analyses. This finding is similar to those from Lawrence et al. [38], where there was a higher proportion of calves with diarrhoea than adults. In contrast to the NZ study, we found that anaemia was more likely to occur in calves compared to adult animals, as were the related signs of lethargy and tachypnoea. However, we also found that the association between parasite load and anaemia was less strong in calves compared to adults, perhaps suggesting that other underlying or undiagnosed conditions may have contributed to the severity of disease in calves. Indeed, data from both studies suggest that mortalities are more common in calves, and anecdotally, calves often present with secondary conditions such as scouring and pneumonia. 

Given the potential impacts of animal age, production type, husbandry practices, host immunity, and vector activity on the clinical manifestations of theileriosis, epidemiological studies of disease outbreaks in other countries, such as those recently reported in the United States, will provide an opportunity to further explore how these factors influence the course of infection and, in the absence of vaccines or chemotherapeutic options, better inform disease management. 

## Figures and Tables

**Table 1:** Number of jobs received from each Australian state between 2006 and 31 December 2022.

State	New South Wales (NSW)	Victoria (VIC)	Queensland (QLD)	Western Australia (WA)	South Australia (SA)	Tasmania (TAS)
No. of jobs received	1457	76	55	10	4	3

**Table 2:** Odds ratio (OR) for calves and beef cattle of significant recorded clinical signs, including the χ^2^ *p*-value and 95% lower and upper Confidence Intervals (CIs).

Clinical Sign	*p*-Value	OR	CI
**Calves vs. adults**			
Lethargy	<0.0001	2.50	1.61–3.90
Tachypnea	<0.0001	5.63	3.11–10.67
Jaundice	0.01	0.59	0.39–0.88
Diarrhoea	<0.0005	2.67	1.56–4.61
Fever	<0.005	1.79	1.22–2.62
Anaemia	<0.0001	2.79	1.95–4.03
Discharge	0.02	2.71	1.12–6.83
Mortality	<0.0005	1.87	1.33–2.63
**Beef vs. dairy cattle**			
Depression	0.04	2.40	1.08–6.46
Tachycardia	0.02	2.58	1.16–6.90
Fever	<0.0001	2.52	1.63–4.03
Abortion	<0.0001	6.40	3.14–15.49
Ill-thrift	<0.0001	0.20	0.06–0.54
Mortality	<0.0001	2.98	2.12–4.23

## Data Availability

The original contributions presented in the study are included in the article/Supplementary Material; further inquiries can be directed to the corresponding author.

## Supplementary Materials

The following supporting information can be downloaded at: https://www.mdpi.com/article/10.3390/pathogens13030253/s1, Video S1 Animation of number and location of Ikeda-positive herds detected every month between 2006 and 2022 in Australia; Video S2: Animation of number and location of Ikeda-positive herds detected every month between 2006 and 2022 in NSW showing Local Land Service District boundaries; Table S1: Contingency table of clinical signs against age (calf, adult, or unknown), farm type (beef, dairy, or unknown), and anaemia level (normal, moderate, or severe).
